# Handheld computers for data entry: high tech has its problems too

**DOI:** 10.1186/1745-6215-8-5

**Published:** 2007-02-20

**Authors:** Tania M Shelby-James, Amy P Abernethy, Andrew McAlindon, David C Currow

**Affiliations:** 1 Department of Palliative and Supportive Services, Flinders University, Bedford Park, South Australia, Australia; 2 Southern Adelaide Palliative Services, Repatriation General Hospital, Daw Park, South Australia, Australia; 3 Division of Medical Oncology, Department of Medicine, Duke University Medical Centre, Durham, North Carolina, USA; 4 Department of Medicine, Flinders University, Adelaide, South Australia, Australia; 5 HealthConnect SA, Department of Health, South Australia, Australia; 6 Cancer Australia, Canberra, Australia

## Abstract

**Background:**

The use of handheld computers in medicine has increased in the last decade, they are now used in a variety of clinical settings. There is an underlying assumption that electronic data capture is more accurate that paper-based data methods have been rarely tested. This report documents a study to compare the accuracy of hand held computer data capture versus more traditional paper-based methods.

**Methods:**

Clinical nurses involved in a randomised controlled trial collected patient information on a hand held computer in parallel with a paper-based data form. Both sets of data were entered into an access database and the hand held computer data compared to the paper-based data for discrepancies.

**Results:**

Error rates from the handheld computers were 67.5 error per 1000 fields, compared to the accepted error rate of 10 per 10,000 field for paper-based double data entry. Error rates were highest in field containing a default value.

**Conclusion:**

While popular with staff, unacceptable high error rates occurred with hand held computers. Training and ongoing monitoring are needed if hand held computers are to be used for clinical data collection.

## Background

The use of handheld computers has been increasing steadily in medicine over the last decade. As a data entry tool, handheld computers have a number of advantages over paper-based data collection including reduced paperwork, transcription errors, time and cost [1,2]. In some clinical settings it has been estimated that the use of a handheld computer can save nurses nearly two hours per day [3]. Handheld computers have been adapted into a variety of research and clinical settings and available software allows integration into many database types. However, studies have not documented the accuracy of data collection using handheld computers. There is an underlying assumption they are better than a paper-based system.

We recently completed a longitudinal prospective randomised controlled trial of 461 palliative patients [4] followed from referral to a specialised palliative care service until death. This trial generated more than 2 million paper-based data entry points. Prior to instituting handheld computers for data collection and entry, we assessed user accuracy. Our aim was to use handheld computers to reduce trial nurse's workload.

## Participants, Methods, and Results

An Ethics Committee-approved comparison study was undertaken with 6 trial forms. Staff undertook training on Compaq Pocket PCs model iPAQ H3950. Research nurses completed a traditional paper-based form that was entered into a database by administrative staff in parallel with the same nurse completing an electronic form on the handheld computer. Data were transferred from the handheld computer using a manual cradle. Results of these two methods were compared for discrepancies; the paper form was considered the gold standard.

A total of 2001 data elements from 29 consecutive trial participants were entered using both electronic and paper-based methods. Error rates for data entry using the handheld computers were 67.5 errors per 1000 fields, much higher than the accepted 10 per 10,000 fields for paper-based double data entry[5]. Error rates were highest in fields containing a default value such as a date.

Staff found the handheld computers easy to use and liked using them as a reference tool however they reported discomfort using them for data collection. As reported elsewhere, [6] we experienced a number of technical problems with uploading and downloading data that caused frustration and dissatisfaction. This may have been reduced using a wireless system.

## Comment

Rarely have evaluations of user accuracy been undertaken when introducing new technologies for data collection. In contrast to the belief that handheld computers would decrease error rates, we found unacceptably high error rates. This finding has implications for the introduction of electronic data collection for research or clinical purposes without an adequate testing phase.

Error rates need to be monitored if handheld computers are to be used both at the time they are introduced, and in an ongoing way.

## Competing interests

The author(s) declare that they have no competing interests.

## Authors' contributions

TSJ carried out conception and design, obtaining funding, acquisition of data; analysis and interpretation of data and drafting of the manuscript. AA carried out conception and design; analysis and interpretation of data and critical revision of manuscript. AM provided technical advice and design of the data base and critical revision of the manuscript. DC participated in the design of the study, interpretation of the data, critical revision of the manuscript and supervision. All authors read and approved the final manuscript.
